# Adherence to *Staphylococcus aureus* Bacteremia Management Recommendations Before, During, and After the COVID-19 Pandemic: Prognostic Implications

**DOI:** 10.3390/antibiotics14060615

**Published:** 2025-06-18

**Authors:** Elizabeth Lorenzo-Hernández, Francisco Rivas-Ruiz, Jorge Fernández-Casañas, Vanesa Puerto-Romero, Maria Dolores Martín-Escalante, Alfonso Del Arco-Jiménez

**Affiliations:** 1 Internal Medicine Department, Costa del Sol University Hospital, A-7, Km 187, 29603 Marbella, Malaga, Spain; jorge.fernandez.casanas.sspa@juntadeandalucia.es (J.F.-C.); vanesa.puerto.sspa@juntadeandalucia.es (V.P.-R.); mdolores.martin.escalante.sspa@juntadeandalucia.es (M.D.M.-E.); 2 Research Unit, Costa del Sol University Hospital, A-7, Km 187, 29603 Marbella, Malaga, Spain; francisco.rivas.ruiz.sspa@juntadeandalucia.es; 3 Infectious Diseases Group, Internal Medicine Department, Costa del Sol University Hospital, A-7, Km 187, 29603 Marbella, Malaga, Spain

**Keywords:** *Staphylococcus aureus*, bacteremia, bloodstream infections, clinical management, pandemic, bundle, quality of care, complications, mortality

## Abstract

**Background/Objectives:** This work aims to assess the evolution in the management of *Staphylococcus aureus* bacteremia (SAB) and the impact of the COVID-19 pandemic on it. SAB is associated with high morbidity and mortality, requiring structured management strategies. The COVID-19 pandemic led to major changes in hospital workflows, potentially affecting the quality of SAB care. **Methods:** We conducted a retrospective per-protocol analysis of SAB episodes at Costa del Sol University Hospital (Marbella, Spain) across three periods: pre-pandemic, pandemic, and post-pandemic. Patients with early mortality or early transfer were excluded. Clinical variables, adherence to recommended management bundles, and outcomes were compared. Demographic characteristics were similar across the analyzed periods. **Results:** The incidence of SAB increased over time, with a notable rise post-pandemic. Key management indicators such as the identification of infection source and appropriate antibiotic therapy showed adherence rates of above 90%, while echocardiography exhibited an adherence rate of above 75% throughout the study. Adherence to the full management bundle was suboptimal, with no significant differences between periods. However, an appropriate treatment duration significantly improved in the post-pandemic group (*p* = 0.038). Mortality at 14 and 30 days was highest during the pandemic period (10.3% and 17.6%, respectively), although differences were not statistically significant. Complications and mortality were more frequent in patients with complete adherence to the bundle (*p* = 0.031). **Conclusions:** Despite stable or improved adherence to certain SAB management measures during the pandemic, mortality and complication rates did not significantly decrease, likely reflecting increased patient severity or healthcare system overload. These findings highlight the need for sustained, multidisciplinary, bedside-based approaches to SAB care, even during public health emergencies. Further research is needed to explore modifiable factors and enhance adherence to evidence-based recommendations.

## 1. Introduction

*S. aureus* bacteremia (SAB) is highly prevalent in our setting, with an estimated incidence of approximately 50 cases per 100,000 person-years [1,2,3,4,5,6,7,8,9,10]. It entails high morbidity and mortality, close to 20–30%, resulting from the bacteremia itself or its complications due to hematogenous spreading. It has been demonstrated that implementing a series of measures following the presentation of bacteremia leads to an improvement in prognosis. These measures include identifying the infection source; ensuring the appropriate duration of treatment for the infection; performing echocardiography; and conducting follow-up blood cultures, typically carried out by infectious disease (ID) experts or members of an Antibiotic Stewardship Program (ASP) [1,2,3,4,6,7,8,9,10,11,12,13,14,15]. However, adherence to these measures is still not at 100% [3]. Despite advances in knowledge, a standardized management of this condition has not yet been achieved. There are still differences among healthcare professionals and institutions [6,8].

Greater efforts were made to diagnose SAB during the pandemic. It became one of several infectious processes considered in differential diagnoses, often leading to delayed recognition and intervention. Some studies suggest that differences in mortality rates across centers may stem from heterogeneous approaches to patient care.

Our institution is a secondary-level hospital with 408 beds. It serves a population of nearly half a million people [16]. Depending on the incidence waves during the pandemic, hospitalizations due to SARS-CoV-2 infection accounted for up to 90% of admissions at certain times, with big implications for hospital reorganization.

The COVID-19 pandemic has had a negative impact on hospital systems, necessitating a reorganization of services and forcing healthcare professionals to work under pressure, uncertainty, and increased stress. This scenario may have led to suboptimal management of non-COVID-19 conditions, such as SAB [1,4,12]. The literature reports cases in which the pandemic contributed to poorer SAB management, including increased mortality [1]. Moreover, the management of SAB is not uniform across hospitals worldwide [2,17]. These differences may have been exacerbated during the pandemic, in the context of a healthcare crisis, for example, due to limited access to complementary diagnostic procedures such as echocardiography or reduced follow-up by ASPs.

However, despite the impact of the COVID-19 pandemic on hospital organization, these measures are primarily educational in nature, and an increasing adherence to the SAB management bundle is expected. Nevertheless, the impact of the pandemic on ASPs and the management of bacteremia remains unclear, as the few available studies on this topic have reported conflicting results [1,4,12]. Given these discrepancies, the present study aims to evaluate the influence of the COVID-19 pandemic on the management of SAB in our center. Specifically, we sought to determine how adherence to evidence-based recommendations evolved over time, and whether these changes had any measurable impact on patient outcomes. The hypothesis was that the COVID-19 pandemic negatively affected adherence to the SAB management bundle, as estimated by comparison with pre- and post-pandemic periods, and that this had an adverse impact by increasing mortality and/or complications.

## 2. Results

A total of 359 episodes of bacteremia were obtained in the study period: 90 episodes in the pre-pandemic period, 122 corresponding to the pandemic phase group, and 137 in the post-pandemic period. To analyze the quality of care, only patients who were able to complete the bundle were selected (those who experienced early mortality or early transfer to other hospitals were excluded), i.e., a per-protocol analysis was conducted. After reviewing the episodes, a total of 276 patients remained: 75 in the pre-pandemic group, 97 in the pandemic period, and 104 in the final group.

Demographic and clinical variables are described in Table 1. The Pitt bacteremia score was significantly higher in the first group. There were no differences in disease acquisition. Regarding the cause of infection, the two most frequent were skin and soft tissue infection (SSTI) and catheter-related.

To assess the management of bacteremia, the main variables measured were the identification and control of the infection source, the performance of follow-up cultures, the use of echocardiography for endocarditis screening, and the appropriate duration of antibiotic treatment. In this regard, source identification was performed in 92% of cases in the pre-pandemic group, 95.9% in the pandemic group, and 96.1% in the post-pandemic group. Appropriate treatment duration was observed in 89.2% of the first group, 90.6% of the second group, and 98.1% of the third group; significant differences were observed between the groups. Echocardiography was performed in 78.7% of the cases in the pre-pandemic group, 78.4% in the pandemic group, and 76% in the post-pandemic group. The adherence to all recommendations was 53.3% in the pre-pandemic group, 52.6% during the pandemic, and 47.1% in the post-pandemic group, with no significant differences between the groups. Additionally, the appropriateness of antibiotic prescription in terms of spectrum and dosage was assessed, both empirically and after blood culture results (Table 2).

The presence of written recommendations in the medical record by the ID expert (and/or the on-call medical team) was evaluated, along with the attending physician’s level of adherence to these recommendations (Table 2).

Regarding the outcome of bacteremia, patients included in the protocol (since those with early mortality were excluded) in the pandemic phase group had a mortality rate of 10.3% at 14 days and 17.6% at 30 days, while in the pre-pandemic group, it was of 8% and 12%, and in the post-pandemic group, it was of 5.8% and 12.6%, respectively. However, no significant differences were found between the groups (Table 3). When analyzing whether adherence to recommendations influenced the occurrence of complications or death at 30 days, it was observed that these outcomes occurred in 27% of patients with full adherence to recommendations, compared to 11.1% with no adherence and 4.3% with partial adherence (*p* = 0.031).

Multivariate analysis identified the Pitt score as a predictor of complications/30-day mortality.

## 3. Discussion

This study investigated the quality of care in the management of SAB in the Costa del Sol University Hospital in Marbella (Málaga, Spain) before, during, and after the COVID-19 pandemic and its effects on SAB management. This is the largest study to observe the impact of the COVID-19 pandemic on the management of SAB [1,4] and one of the few studies evaluating real-world outcomes related to adherence to evidence-based management recommendations for SAB [10,18].

In our institution, after notification from the Microbiology Department regarding the positivity of blood cultures, protocol-based recommendations are provided to the attending physician as unsolicited advice of an ASP for all non-intensive-care-unit patients, primarily in a verbal manner, all of this with an educational rather than an imposing approach [2,6,9]. The COVID-19 pandemic brought about significant organizational changes in our hospital. At certain times (primarily during the first and third waves), the hospital’s activity focused on caring for 90% of hospitalized patients with SARS-CoV-2 infection. Nevertheless, advice from the ID expert and/or the on-call medical team was maintained.

The incidence of SAB progressively increased over the study period, although no statistically significant differences were observed between the groups. These findings are consistent with those reported in previous studies [19,20,21,22]. However, other authors described a decrease in SAB incidence during the pandemic period [1,4]. In our cohort, a 50% increase in SAB incidence was observed in the post-pandemic group compared to the pre-pandemic group. This notable rise may reflect changes in healthcare dynamics, infection control practices, or patient characteristics after the critical phase of the COVID-19 pandemic. To our knowledge, no previous studies have specifically assessed trends in SAB incidence beyond the peak of the pandemic, highlighting the need for further investigation in this area.

Regarding the characteristics of the included patients, no major differences were found compared to other studies [1,4,6,13]. However, the high rate of nosocomial infection, approximately 50%, was lower than the reported in the literature [4], despite the sample selection criteria. By excluding patients with early mortality or early transfer, the proportion of community-acquired or healthcare-associated infections was reduced. Nevertheless, it remained lower than that observed in other centers. In most published studies, the primary source of infection was catheter-related [1,2,4]. Interestingly, the rate of catheter-associated infection was lower in the pandemic group compared to the other periods, which may have been influenced by heightened awareness and adherence to hygiene practices during this time [4,22]. Additionally, other articles examining the impact of the pandemic on infections in general reported an increase in MRSA-related SAB [23,24,25], a trend also observed in our cohort. This tendency appeared to persist in the post-pandemic period, in line with the ongoing rise of antimicrobial resistance, although no statistically significant differences were found.

Regarding adherence to recommendations, no significant differences were observed between the groups in the main management measures when comparing the pre-pandemic and pandemic periods, consistent with the findings of Böing et al. [4]. While differences in adherence to specific measures were not statistically significant, certain positive trends were evident, particularly in the identification of the infectious source and the duration of antibiotic therapy. Nonetheless, the individual impact of each component on patient outcomes remains unclear [2,6,9]. Some evidence suggests that follow-up blood cultures are crucial, as persistent bacteremia beyond 48 h has been associated with a significant increase in mortality [17,26]. Furthermore, this study is the only one reporting that adherence rates above 75% had a limited impact on mortality [27]. Although the overall decrease in mortality was not statistically significant, the only factor likely contributing to the observed reduction was the appropriate duration of antibiotic therapy, for which statistically significant differences were indeed identified.

Although the overall adherence to the bundle did not show significant differences between the three periods, there was a trend toward greater implementation of certain key measures—such as early initiation of antibiotic treatment (<24 h) or appropriate empirical dosage—in the groups with a higher proportion of patients with sepsis at diagnosis (pandemic and post-pandemic). Interestingly, higher adherence to bundle management was observed in patients who experienced complications or died, possibly reflecting a tendency to apply more intensive care measures in cases perceived as more severe. Nonetheless, adherence to bundle management was higher in all aspects, including complete bundle adherence, except for the performance of echocardiography, than in the pre-intervention group of Casalini et al. [13]. The lower rate of echocardiogram performance during the post-pandemic period was likely due to a reduced perception of the severity of the infection and a greater degree of relaxation compared to the acute phase of the pandemic. During the COVID-19 pandemic, a reduction in admissions for scheduled procedures was noted, implying a higher proportion of acutely ill hospitalized patients [28], which may have contributed to greater adherence to bundle management compared to the most recent period. This observation could suggest that, in clinical practice, cases with higher perceived severity might have received more intensive care and closer adherence to recommendations, which could reflect increased awareness or a more responsive clinical approach to more severe cases. No differences were observed in the median Pitt score, so this consideration must be taken with caution, although the range values are higher in the two later groups.

It was also observed that during the pandemic period, there was a high level of adherence to the bundle, despite the low rate of written recommendations, which could be partially explained by increased awareness among healthcare professionals regarding infectious diseases [4].

In the main studies evaluating differences in the quality of care for SAB, the distinction lies between those who maintained their pre-existing workflows (Böing et al.) [4] and those who significantly reduced the number of consultations due to hospital overload (Arientová et al.) [1], with our results being more closely aligned with the former. Another study implemented a quality control program for the management of SAB during the pandemic period, achieving high adherence to the recommended measures but without a reduction in mortality. This outcome was likely influenced by a higher Charlson Comorbidity Index and other factors such as bacterial coinfection, patient isolation, and healthcare system and staff overload (Jakoby et al.) [29]. Although adherence to the recommendations in our population remains suboptimal, studies specifically assessing the prognostic impact of bundle implementation in the management of SAB—such as the work by Escrihuela-Vidal et al.—report an overall low adherence rate, around 18%. This low adherence is mainly attributed to insufficient source identification and low rates of follow-up blood cultures, partly explained by the heterogeneity of the participating centers [6].

A non-significant increase in mortality was observed during the COVID-19 pandemic, despite better adherence to measures. This increase in mortality may be attributed to unmeasured factors (overload). However, slightly older age was observed in this group compared to the others, a clear risk factor [4,6,7,9,26]. Additionally, a higher number of cases caused by MRSA was observed [9,26]. Over time, a greater occurrence of complications was noted, which may be influenced by various factors beyond the baseline characteristics of the samples.

Few studies evaluate adherence to recommendations within a real-life/retrospective setting [10,18,29]. Other studies report higher adherence rates within prospective programs, with potential selection and observer biases [2,8,13,27].

In several institutions, initiatives are being implemented to improve adherence to recommendations that enhance the prognosis of SAB, such as the introduction of real-time automated alerts for bacteremia [3] or a multidisciplinary follow-up approach, which includes pharmacists in the development of a real-time algorithm, without substituting the ID consultation [7,30,31]. However, these studies have not compared these approaches with direct bedside ID expert consultation, a strategy that has been shown to be more effective than alternative methods like telephone alerts [32]. Nevertheless, the healthcare pressure experienced during this period may be reflected in the reduced number of written recommendations recorded, which is a measure that has been shown to increase adherence to the bundle [15]. This may have influenced the outcomes. Furthermore, it remains crucial to identify other modifiable factors that could influence both adherence to recommended measures and the overall prognosis of patients [33].

Based on the analysis of our results and until the publication of this article, we have transitioned from passive oversight to a more proactive follow-up approach. Our institution has recognized the need for a more structured approach to the management of SAB. This includes mandatory follow-up, source identification, follow-up cultures, and echocardiography, in addition to providing antibiotic guidance, all aimed at improving the prognosis of patients under our care. So, now is the ID expert who actively requests these tests. Also, this individual provides written recommendations directed to the responsible physician(s). As a potential future intervention, given that adherence to the management bundle for SAB has shown opportunities for improvement, the implementation of a Failure Modes and Effects Analysis (FMEA) is proposed to identify vulnerabilities within the process and to design proactive strategies aimed at optimizing case management. Incorporating this structured methodology into clinical practice may enhance compliance with evidence-based recommendations and ultimately contribute to reducing complication rates and mortality associated with SAB.

The main limitations of this study were its single-center nature and retrospective design. It should be noted that the nature of the design did not contemplate the calculation of a sample size for the detection of differences in both groups, so the differences may be underestimated. Although the census-based methodology attempts to control potential confounding factors, it is possible that other factors influencing the results were not measured. Furthermore, mortality was estimated in a crude manner, without distinguishing whether it was directly attributable to the bacteremia itself or other concurrent causes. This study has a publication delay due to the impossibility of extracting the data until definitive information is available in the digital clinical history of our hospital, which has a 1-year lag.

## 4. Materials and Methods

### 4.1. Study Design

This was a retrospective observational study that included non-pediatric patients (≥14 years old) with *S. aureus* bacteremia confirmed by blood cultures, as recorded in the bacteremia registry of Costa del Sol University Hospital (Marbella, Málaga, Spain). The analysis focused on cases identified during the acute phase of the COVID-19 pandemic in Andalusia, as defined by the official regional COVID-19 reports [33]. These cases were compared with two control periods of equal length—one preceding the pandemic (from 1 February 2018 to 29 February 2020) and one following it (from 1 April 2022 to 30 April 2024). Demographic, clinical, and outcome-related variables were extracted from the electronic medical records. In cases of persistent bacteremia, only a single episode per patient was considered for analysis.

The Costa del Sol Research Ethics Committee approved this study with the code 003_mar25-PI—ProaCovid on 27 March 2025.

### 4.2. Participants

Inclusion criteria were all patients older than 14 years (census-based study) treated at the Costa del Sol University Hospital in Marbella (Málaga, Spain), who developed SAB, from 1 February 2018 to 30 April 2024. All patients who did not complete treatment at our center or in the public hospital network of Andalusia were excluded. Also, all patients with early mortality, within the first 5 days from the onset of bacteremia, were excluded. This is because they may not have completed two of the bundle items, namely control blood cultures and the performance of an echocardiogram.

### 4.3. Definitions

Healthcare-related acquisition was defined as that in which patients resided in a nursing home or received care at dialysis centers or outpatient clinics, and nosocomial acquisition was defined as that in which bacteremia occurred more than 48 h after admission. Comorbidities were assessed using the Charlson Comorbidity Index, while prognosis was evaluated with the Pitt bacteremia score and the McCabe and Jackson classification.

The source of infection was identified based on clinical documentation in the patient’s medical history, microbiological criteria (such as the isolation of *S. aureus* from other clinical specimens), or a combination of both. Antibiotic therapy was categorized as empirical or targeted depending on whether it was initiated before or after the availability of blood culture results, respectively. Treatment adequacy was evaluated in terms of antimicrobial spectrum and dosage, in accordance with current guidelines for the latest review of SAB management [26].

Compliance with the bundle was defined by receiving an adequate duration of therapy, performing follow-up blood cultures 48 to 72 h after initiating appropriate antimicrobial treatment, and conducting an echocardiogram when clinically indicated (such as in cases of individual risk factors, persistent bacteremia, complicated bacteremia, community-acquired infection, or unknown source). Persistent bacteremia was defined as the presence of *S. aureus* isolated from follow-up blood cultures obtained 48 to 72 h after starting appropriate therapy. The appropriate duration of treatment was considered to be 14 days for uncomplicated bacteremia, with extended therapy recommended for complicated cases or when the infection source or complications necessitated prolonged treatment [2,26,27].

### 4.4. Microbiological Identification Techniques

Microbiological identification was carried out directly from positive blood cultures using matrix-assisted laser desorption/ionization time-of-flight mass spectrometry (MALDI-TOF, Bruker Daltonics, Billerica, MA, USA). After confirming *S. aureus* identification, polymerase chain reaction (PCR) was performed directly on the positive blood culture for the rapid detection of methicillin-resistant genes (mecA/mecC) using the BD MAX StaphSR Assay (BD, Billerica, MA, USA). Antimicrobial susceptibility testing was conducted via the turbidimetric method with the Vitek 2 system (Biomérieux, Marcy-l’Étoile, France). The identification of *S. aureus* from additional clinical specimens (e.g., catheters and respiratory samples) was also achieved by MALDI-TOF mass spectrometry. The detection of SARS-CoV-2 was performed using various commercial PCR kits on nasopharyngeal swab specimens.

### 4.5. Main Study Variables

The main variables to determine adherence to the SAB bundle over time were the identification of the infection source, the appropriate duration of antibiotic therapy, performing echocardiography, and follow-up blood cultures. Additionally, mortality at 14 and 30 days from the first positive blood culture was assessed, and the development of complications, defined as endocarditis, osteoarticular infections, myositis, aortitis, lung abscess, central nervous system abscess, abscesses in other locations, or other related conditions, were measured.

### 4.6. Statistical Analysis

We performed a descriptive analysis using measures of central tendency, dispersion, and position for quantitative variables, and frequency distribution for qualitative variables. For the bivariate analysis of each dichotomous outcome variable of interest, the Chi-square test (or Fisher’s exact test when appropriate) was used for qualitative independent variables. We performed an ANOVA test (or the Kruskal–Wallis test in cases of non-normal distribution) for quantitative variables. We constructed a multivariate logistic regression for the outcome complications or 30-day mortality, as well as for compliance with all recommendations and adherence to individual recommendations. We used the forward stepwise method with the independent variables of interest (study period, age, methicillin resistance status of the isolate, and categorized Pitt score (0, 1–2, ≥3). For all analyses, statistical significance was set at *p* < 0.05. Data were analyzed using SPSS version 28 software.

## 5. Conclusions

SAB is a complex and multifaceted infection, often leading to significant morbidity and mortality. Its management needs the involvement of a dedicated team of experts at each hospital, with a focus on providing guidance and continuous follow-up, preferably at the patient’s bedside. However, public health emergencies, such as the COVID-19 pandemic, can compromise the implementation of these essential measures, as resources and efforts are often redirected toward managing the surge of infected patients. The adherence to established recommendations varies across healthcare centers, and while our institution demonstrated an acceptable level of adherence to certain components of the management bundle, this adherence was higher during the pandemic period. Despite these improvements in adherence, no statistically significant reductions in mortality were observed; in fact, mortality rates increased during the pandemic. These findings emphasize the importance of sustaining evidence-based management strategies to improve outcomes in patients with SAB, even under crisis conditions. They also underscore the need for continued implementation of evidence-based practices aimed at improving patient outcomes. Further studies with larger sample sizes are necessary, to explore potential risk factors for adverse outcomes and refine strategies to enhance the management of SAB.

## Figures and Tables

**Table 1:** Demographic and clinical characteristics.

		Pre-Pandemic Period	Pandemic Period	Post-Pandemic Period	*p*
Total		75	97	104	-
Sex (male); N (%)		57 (76)	69 (71.1)	78 (75)	0.940
Age; Mean years (SD)		61.6 (17.4)	66.1 (15.4)	63.8 (16.9)	0.210
Charlson Comorbidity Index; Median (P25–P75, range)		2 (1–4.0, 0–8)	2 (0–3.5, 0–8)	2 (1–4, 0–12)	0.380
Pitt score; Median (P25–P75, range)		1 (0–2, 0–6)	0 (0–2, 0–14)	0 (0–1, 0–8)	0.025
Mccabe; n (%)	Not fatal	32 (42.7)	45 (46.4)	33 (31.7)	0.500
	Quickly fatal	20 (26.7)	31 (32)	44 (42.3)	
	Ultimately fatal	23 (30.7)	21 (21.6)	27 (26)	
Sepsis at diagnosis, n (%)		21 (28.4)	31 (32)	37 (35.9)	0.570
Bacteria; n (%)	MSSA	71 (94.7)	90 (92.8)	91 (87.5)	0.190
	MRSA	4 (5.3)	7 (7.2)	13 (12.5)	
Acquisition; n (%)	Community	26 (34.7)	26 (26.8)	30 (28.8)	0.640
	Related to healthcare	19 (25.3)	21 (21.6)	23 (22.1)	
	Nosocomial	30 (40)	50 (51.5)	51 (49)	
Source of infection; n (%)	Catheter	24 (32)	21 (21.6)	32 (30.8)	-
	SSTI	18 (24)	26 (26.8)	18 (17.3)	
	Respiratory	12 (16)	15 (15.5)	13 (12.5)	
	Endocarditis	4 (5.3)	8 (8.2)	4 (3.8)	
	Osteoarticular	3 (4)	5 (5.2)	13 (12.5)	
	Abdominal	1 (1.3)	6 (6.2)	4 (3.8)	
	Urinary	3 (4)	5 (5.2)	12 (11.5)	
	Others	1 (1.3)	1 (1)	0	
	Primary	9 (12)	10 (10.3)	8 (7.7)	

n: total number of patients in that category. SD: standard deviation. P25: 25th percentile. P75: 75th percentile. MSSA: Methicillin-sensitive *S. aureus*. MRSA: Methicillin-resistant *S. aureus*. SSTI: skin and soft tissue infection.

**Table 2:** Management of the bacteremia.

		Pre-Pandemic Period	Pandemic Period	Post-Pandemic Period	*p*
Adequate empirical therapy, n/total (%)		56/72 (77.8)	81/94 (86.2)	80/97 (82.5)	0.370
Adequate empirical dosage, n/total (%)		51/71 (71.8)	77/86 (89.5)	83/97 (85.6)	0.009
Early empirical therapy (before 24 h), n/total (%)		57/73 (78.1)	90/97 (92.8)	90/104 (86.5)	0.020
Adequate targeted therapy, n/total (%)		69/73 (94.5)	91/96 (94.8)	100/103 (97.1)	0.640
Adequate targeted dosage, n/total (%)		66/74 (89.2)	87/96 (90.6)	101/103 (98.1)	0.040
Infection source search, n/total (%)		69/75 (92.0)	93/97 (95.9)	99/103 (96.1)	0.400
Adequate duration, n/total (%)		66/74 (89.2)	87/96 (90.6)	101/103 (98.1)	0.038
Echocardiogram, n/total (%)		59/75 (78.7)	76/97 (78.4)	79/104 (76.0)	0.890
Control blood cultures, n/total (%)		48/75 (64.0)	58/97 (59.8)	62/104 (59.6)	0.810
Compliance with all recommendations, n/total (%)		40/75 (53.3)	51/97 (52.6)	49/104 (47.1)	0.640
Written recommendations		34 (45.3)	39 (40.2)	58 (55.8)	0.080
Adherence to the expert recommendations, n/total (%)	No adherence	7 (20.6)	4 (10.3)	7 (12.1)	0.310
	Partial	4 (11.8)	5 (12.8)	14 (24.1)	
	Total	23 (67.6)	30 (76.9)	37 (63.8)	

n: total number of patients in that category.

**Table 3:** Outcomes.

		Pre-Pandemic Period	Pandemic Period	Post-Pandemic Period	*p*
Complications at 30 days	Total	72	91	103	-
	None	60 (83.3)	79 (86.8)	85 (82.5)	
	Endocarditis	5 (6.9)	3 (3.3)	6 (5.8)	
	Osteoarticular	0	1 (1.1)	2 (1.9)	
	Abscess	2 (2.8)	1 (1.1)	6 (5.8)	
	Pulmonary embolism	3 (4.2)	2 (2.2)	2 (1.9)	
	CNS embolism	0	3 (3.3)	1 (1)	
Complications/death at 30 days, n (%)		17/74 (23.0)	25/95 (26.3)	35/104 (33.7)	0.260
Death at 14 days, n (%)		6/75 (8.0)	10/97 (10.3)	6/104 (5.8)	0.490
Death at 30 days, n (%)		9/75 (12.0)	17/95 (17.9)	13/103 (12.6)	0.460

n: total number of patients in that category. CNS: central nervous system.

## Data Availability

The data that support the findings of this study are available upon request from the corresponding author.

## Abbreviations

The following abbreviations are used in this manuscript:

ASP	Antibiotics Stewardship Program
FMEA	Failure Modes and Effects Analysis
ID	Infectious disease
MRSA	Methicillin-resistant *S. aureus*
PCR	Polymerase chain reaction
SAB	*S. aureus* bacteremia
